# 430. Tumor microbiota and tumor-infiltrating T cells in the tumor microenvironment of colorectal cancer

**DOI:** 10.1093/ofid/ofae631.144

**Published:** 2025-01-29

**Authors:** Satoko Ugai, Tomotaka Ugai, Shuji Ogino

**Affiliations:** Harvard T.H. Chan School of Public Health, Brookline, MA; Brigham and Women's Hospital, Brookline, Massachusetts; Brigham and Women's Hospital, Brookline, Massachusetts

## Abstract

**Background:**

Recent studies indicate that the gut microbiota influences the immune cell infiltrates in colorectal cancer. For example, a study has shown that the amount of *Fusobacterium nucleatum* in colorectal carcinoma has been inversely associated with CD3^+^ cell density in tumor. Evidence also indicates that *Bacteroides fragilis* may suppress the adaptive T cell response. Based on such biological evidence, we hypothesized that T cell infiltrates might differ by the colorectal tumor tissue microbiota.

**Methods:**

We conducted 16s rRNA sequencing to identify the abundance of 1,518 microbiome species in 861 colorectal cancer patients in the Nurses’ Health Study and the Health Professionals Follow-up Study. Multiplex immunofluorescence combined with digital image analysis and machine learning algorithms was used to measure various T-cell subsets, such as CD3, CD4, and CD8. Spearman correlation test was performed to investigate the association between the abundance of gut microbiota and CD3^+^, CD3^+^CD4^+^, and CD3^+^CD8^+^ T-cell densities in the overall tumor region. For the validation, we used tumor microbial data by qPCR.

**Results:**

The strongest negative association was observed between the genus *Clostridium* and CD3^+^CD4^+^ T-cell density (rho = -0.13, p=8.27E-05). *Bacteroides fragilis* had a statistically significant negative association with CD3^+^ T-cell density (rho = -0.10, p = 0.0026) and CD3^+^CD4^+^ T-cell density (rho = -0.12, p < 0.001). *Fusobacterium nucleatum* also had a significant negative association with CD3^+^ T-cell density (rho = - 0.078, p = 0.021) and CD3^+^CD4^+^ T-cell density (rho = -0.11, p = 0.0011). These findings were validated by analyses using qPCR data.

**Conclusion:**

We identified tumor microbiota, the abundance of which was inversely associated with tumor-infiltrating T cells in the tumor microenvironment of colorectal cancer. The strongest negative association was observed between *Clostridium* and CD3^+^CD4^+^ cell density. The abundance of *Bacteroides fragilis* and *Fusobacterium nucleatum* correlated negatively with the density of CD3^+^ and CD3^+^CD4^+^ cells in colorectal carcinoma tissue. Our findings provide new evidence for microbial-immune interactions in the tumor microenvironment.

**Disclosures:**

**All Authors**: No reported disclosures

